# First Report of Prevalence of Blood-Borne Viruses (HBV, HCV, HIV, HTLV-1 and Parvovirus B19) Among Hemophilia Patients in Afghanistan

**DOI:** 10.1038/s41598-019-43541-8

**Published:** 2019-05-13

**Authors:** Sayed Hamid Mousavi, Niloofar Khairkhah, Tina Delsouz Bahri, Ali Anvar, Alireza Azizi Saraji, Bita Behnava, Seyed Moayed Alavian, Ali Namvar

**Affiliations:** 1 Molecular Diagnostic Divisions, Iranian Comprehensive Hemophilia Care Center, Tehran, Iran; 2 Department of the Biochemistry, Faculty of medicine, Kateb University, Kabul, Afghanistan; 3 Afghanistan National Charity organization for Special Diseases (ANCOSD), Kabul, Afghanistan; 4 Department of Microbiology, Faculty of Medicine, Shahid Beheshti University of Medical Sciences, Tehran, Iran; 5 Baqiyatallah Research Center for Gastroenterology and Liver Diseases, Baqiyatallah University of Medical Sciences, Tehran, Iran; 6 Department of Molecular Medicine, School of Medicine, Qazvin University of Medical Sciences, Qazvin, Iran

**Keywords:** Viral infection, Infectious-disease diagnostics

## Abstract

Blood-borne viruses including Hepatitis B and C, HIV, HTLV-1 and parvovirus B19 are still a factor of concern, especially for hemophilia patients. Although the safety of the blood supply continues to improve worldwide, the blood supply system in Afghanistan was damaged by many years of conflict and political instability. To date, there are few studies focused on the prevalence of blood-borne viruses in hemophilia patients. This study is first to investigate the prevalence of five blood-borne viruses in Afghanistan hemophilia patients in four cities including Kabul, Herat, Mazar-i-Sharif and Jalal Abad. A total of 80 hemophilia male patients were screening for the presence of five transfusion-transmitted viruses using ELISA and PCR. Data obtained showed 2.5% seropositivity for HBV, 8.75% seropositivity for HCV, and 91.25% seropositivity for parvovirus B19. None of the patients were positive for HIV and HTLV-1 and the prevalence of HCV was higher in older patients rather than younger patients. This finding, the first to report in Afghanistan, shows a high prevalence of parvovirus B19 in Afghanistan hemophilia patients and implementation of highly sensitive screening is necessary.

## Introduction

Of the various types of hemophilia, hemophilia A and hemophilia B are genetic disorders caused by coagulation factor deficiencies of factor VIII and factor IX, respectively. As hemophilia A and B are X-linked recessive disorders, it mainly affects males^1^. The most widely used hemophilia classification is based on deceased factors clotting activity, with persons >5% factors, as mild hemophilia, with persons 1–5%, as moderate hemophilia and with persons <1% factor, as severe hemophilia^2^. The lower the clotting factor activity level, the more frequent spontaneous bleeding episodes happen.

The incidence of hemophilia A is approximately 1:5000 male births, whereas the incidence of hemophilia B is 1:30,000 male births, although reports vary widely between countries^3^. After many years of conflict and political instability, Afghanistan’s health system remains among the very poorest in the world. Therefore, there are no accurate reports of the incidence of hemophilia in this country.

Modern treatment for hemophilia bleeding disorder started in the 1970s, with the wide availability of safe plasma-derived coagulation factors and widespread adoption of home-administered replacement therapy led to the early control of hemorrhages. The optimistic perception of hemophilia changed at a time when plasma-derived concentrates manufactured from pooled plasma obtained from thousands of donors were invariably contaminated with hepatitis B, C virus and HIV and many patients exposed became infected^4^. Consequent to widespread blood-borne virus transmission, the need for improved safety of treatment became crucial for the hemophilia community. The most important advance in this field was represented by recombinant gene technology which enabled the highly purified development of recombinant coagulation factors. So the evolution in the manufacturing process of recombinant factors during the last few years minimized the risk of pathogen transmission and improved the treatment and quality of life of hemophilia patients^5^.

Three pathogens are considered for most cases of acquired blood-borne infection: human immunodeficiency virus (HIV), hepatitis B virus (HBV) and hepatitis C virus (HCV)^6^. In addition, different viruses such as HTLV and parvovirus B19 have been reported in hemophiliacs.

Afghanistan has a poor health infrastructure and accurate HIV awareness and knowledge among Afghans is low. Thus, a concentrated HIV epidemic may soon ensue due increase in high-risk behavior and intravenous drug use^7^. In one cross-sectional study included 464 adult injection drug users (IDUs) in Kabul, Afghanistan, from June 2005 through June 2006, the prevalence of HIV was 3.0%(95% confidence interval [CI] 1.7–5.1%). Risky behavior, such as paying women for sex (76.2%), sharing syringes (50.4%), and having male-to-male sex (28.3%), were common among male IDUs (N = 463). The high prevalence of risky behavior in Kabul indicates the very high risk for an HIV epidemic^8^. In another study of 623 IDUs in three cities of Afghanistan including Herat, Jalalabad and Mazar-i-Sharif, the prevalence of HIV was 1.8% (95% CI 0.88–3.2%) which all HIV cases were detected in Herat. Risky behavior was also common in this study including needle sharing in the last 6 months (30.2%), male-to-male sex (23.1%), and paying females for sex (50.4%)^9^.

The prevalence data for hepatitis C virus (HCV) and hepatitis B surface antigen (HBsAg) is also available in Afghanistan. In thirty-one studies, consisting the data of 132500 individuals for HCV and 132981 individuals for HBV, the prevalence was 1.1% for HCV and 1.9% for HBV in all available Afghanistan population^10^. In one another study of 464 IDUs in Kabul, the prevalence of HBsAg and HCV were 6.5% (95% CI 4.2–8.7%) and 36.6% (95% CI 32.2–41.0%), respectively^8^. In another study of 623 participants, the prevalence of HCV, and HBsAg were 36.0% (95% CI: 33–41%), and 5.8% (95% CI: 3.9–7.6%) which the highest HCV prevalence was found in Herat and the highest HBV prevalence were detected in Jalalabad. Therefore, HIV, HCV and HBV prevention programs are urgently needed in Afghanistan and regional variations should be considered in programming to prevent transmission of blood-borne disease.

Yet in Afghanistan, there are no accurate reports of prevalence of blood-borne viruses in hemophilia patients. In the present paper, we determine the prevalence of transfusion-transmitted diseases including HIV, HCV, HBV, HTLVI and parvovirus B19 among 80 Afghanistan hemophilia patients.

## Result

A total of 80 hemophilia male patients screened in this study. The age range was 2 to 38 years with a mean age of 13.66 (SD 8.95). Patients were classified based on clotting factor level in their blood into three categories: 51 patients (63.75%) had severe hemophilia, 23 patients (28.75%) had moderate hemophilia and six patients (7.50%) had mild hemophilia. Also, thirty-eight of patients (54.28%) had blood transfusion records (Table 1).

**Table 1:** Demographic data of population study.

Area	Sample Size	History of blood transfusion (No. of samples)	Age, mean ± SD (years)	Hemophilia severity levels^*^(No. of samples)
Kabul	31	17 (54%)	11.91 ± 7.93	Severe: 19 Moderate: 9 Mild: 3
Herat	18	11 (61%)	14.80 ± 9.45	Severe: 13 Moderate: 4 Mild: 1
Mazar-i-Sharif	19	5 (26%)	13.57 ± 8.10	Severe: 11 Moderate: 6 Mild: 2
Jalal Abad	12	5 (41%)	16.58 ± 11.76	Severe: 8 Moderate: 4 Mild: 0
Total	80	38 (47%)	13.66 ± 8.95	Severe: 51 Moderate: 23 Mild: 6

^*^Severe: <1 IU/dl or <1% of normal, Moderate: 1–5 IU/dl or 1–5% of normal, Mild: 5–40 IU/dl or 5–40% of normal.

Two hemophilia patients (2.5%, 95% CI: 0.003–0.087) were found to be HBsAg seropositive, whereas HCV Ab was detected in seven patients (8.75%, 95% CI: 0.035–0.172). Parvovirus B19 IgG was found in seventy-three patients (91.25%, %, 95% CI: 0.827–0.964). All 80 patients were negative for HIV and HTLV-1.

A positive DNA/RNA polymerase chain reaction results can be indicated as active/recent infections. The molecular assay shows HBV DNA in two Patients (2.5%, 95% CI: 0.003–0.087), HCV RNA in seven (8.75%, 95% CI: 0.035–0.172) and parvovirus B19 in thirty-five hemophilia patients (43.75%, 95% CI: 0.326–0.553).

There was a significant association between HCV RNA positive test and hemophilia age groups (<0.0001). Most persons born before 1991 were likely to have been infected. Also for HCV infected patients, there was a significant association with the history of blood transmission (0.049). Prevalence of parvovirus B19 DNA was higher in severe hemophilia patients but the difference failed to reach statistical significance level (0.246) (Table 2).

**Table 2:** Distribution of HCV and parvovirus B19 among Afghanistan hemophilia patients(molecular-based assay results).

	HCV Positive (8.75%)	HCV negative (91.25%)	*P* Value	CI (95%)	Parvovirus B19 Positive (43.75%)	Parvovirus B19 negative (56.25%)	*P* Value	*CI* (*95%*)
Age 13.66 (±8.95)			<0.0001	0.003 to 0.177			0.741	0.435 to 1.686
1–25	1	69			30	40		
26–38	6	4			5	5		
Severity of hemophilia			1.000	0.294 to 6.871			0.246	0.801 to 2.522
severe	5	46			25	26		
Mild and moderate	2	27			10	19		
History of blood transfusion			0.049	0.880 to 52.64			0.504	0.499 to 1.375
Yes	6	32			15	23		
No	1	41			20	22		

## Discussion

Transfusion-transmissible infections classified as viral, bacterial and parasitic infections, are an emergent public health problem in different parts of the world especially in areas where blood donor screening practices are weak and the prevalence of parenterally transmitted infections between blood donors is high^11^. As most of the world’s hemophilia population exists in nations with limited medicinal resources and they are unable to obtain virally inactivated clotting products, many patients are treated with locally supplied blood and its components only. Thus, transfusion-transmitted infections such as Hepatitis B virus, Hepatitis C virus and Human Immunodeficiency virus remain a public health problem of hemophilia patients^12^.

The present study aimed to determine the prevalence of HBV, HCV, HIV, parvovirus B19 and HTLV-1 infections among 80 hemophilia male patients in Kabul, Herat, Mazar-i-Sharif and Jalal Abad, Afghanistan. The prevalence of HBsAg and anti-HCV was 2.5% (2/80) and 8.75% (7/80), respectively. All of the 80 hemophilia patients were negative for HIV and HTLV-1 antibody.

The prevalence of HBV infection in hemophilia patients in this study was 2.5%. Both patients with positive HBsAg having severe hemophilia were from Kabul and also have blood transfusion records. Thus, they might be infected by transfusion of contaminated blood. This study seems to be in agreement with all blood donors (with 1.76% HBsAg seropositivity) tested by Central Blood Bank Kabul during the years 1989–2005^10^. The prevalence of HBsAg in Iranian hemophilia patients varies from 0% to 1.6% in most cities^11,13,14^ and 4.9% in Zahedan^15^. It seems that vaccination against HBV infection in all newborns and high-risk groups in Iran and mandatory anti-HBV screening of blood and blood products may be the key to effective control of the infection in hemophilia patients.

The prevalence of HCV in blood donors has been reported 0.14% in Iran^16,17^, and 3.01% to 4.99% in Pakistan^18^. The prevalence of HCV in Iranian hemophilia patients varies from 51% to 80.5% among different cities^13,16,19^. Lower prevalence of HCV in Shiraz, Iran (with 15% HCV seropositivity) can be explained by the stringent policy and guidelines for accurate HCV screening and blood product usage^14^. Also because of compulsory anti-HCV screening in blood units since 1996, the transmission of HCV in Iranian hemophilia patients has reduced remarkably. Such that there was no HCV detected in Iranian hemophilia patients born in Shiraz from 2001 to 2010^20^. The anti-HCV prevalence in Pakistanis hemophilia also varies from 25% in children in Peshawar^21^ to 56% in Lahore^22^. So, the prevalence rate of HCV among Afghanistan hemophilia patients in this study seems to be much lower (8.75%). The low prevalence rate of HCV in this study in comparison with Iran and Pakistan might be in view of the fact that the average age of the participants was low (with the mean age of 13.66). Since in 1980–1990, plasma-derived factor replacement products were contaminated by HIV and HCV, we expect older people to be more infected^23^. All HCV seropositive patients except for one (who was a 2-year-old child with blood transfusion record) were over 25 years old. So, conduct of a nationally-representative population-based survey and mandatory anti-HCV screening in blood banks is recommended to provide a better estimate of HCV prevalence.

All hemophilic patients in this study escaped HIV infection. This is in agreement with hemophilia population in Pakistan^12,24^ and most cities in Iran^14,16^. Low seropositivity of HIV in these populations could be as a result of screening of donated blood units since 1990^20^.

HTLV-1, the first retrovirus identified in human, is also a risk factor for hematologic disorders. Basically, HTLV-1 has a higher prevalence in blood disorders than the general population^25^. So All hemophilic patient in this study were investigated for presence of HTLV-1 which was all negative. This is in agreement with the low prevalence of HTLV-1 and associated disease in Pakistan and other Asian countries except for Japan and Iran^26^. The prevalence of HTLV-1 infection among the general population in Khorasan province, a known endemic region for HTLV-1 in Iran, varied from 1.66% to 7.2%^27–29^. According to one study conducted on 108 hemophilic patients in the Sothern Khorasan province, the seroprevalence of anti-HTLV-1 was 3% which could cause significant health problems and put this population at increased risk.

Human parvovirus B19 was first discovered when a plasma sample from asymptomatic blood donors showed a false-positive result for HBV^30^. Although parvovirus B19 normally spread via the respiratory, it can also transmit via plasma-derived products. Parvovirus B19 infection caused various clinical symptoms among children and adults. The most common parvovirus B19 manifestation among children is a slapped cheek rash on the face causing fifth disease and polyarthropathy among adults^31,32^. Parvovirus B19 in those patients with underlying hemolytic disorders like hemophiliacs may develop into a transient aplastic crisis^32^. In addition, patients with parvovirus B19/HIV co-infection can also develop pure red blood cell aplasia. This subject shows the importance of screening and monitoring blood donors for parvovirus B19.

This study is the first to investigate the prevalence of parvovirus B19 in Afghanistan. The seroprevalence of parvovirus B19 IgG was 91.25% with 43.75% PCR positivity among Afghanistan hemophilia patients, which is higher compared to hemophiliacs in Shiraz, Iran (with 74% seropositivity among 180 patients)^33^. Basically, studies in different countries demonstrated a higher seroprevalence of parvovirus B19 among hemophilia patients. For instance, in one study of 40 hemophilia patients in Japan, 100% of patients were positive for parvovirus B19 IgG and 7/5% of IgG-positive serum samples contained parvovirus B19 DNA^34^. The seroprevalence of parvovirus B19 in Asian blood donors was found to be 25–40%^35,36^.

As parvovirus B19 can be transmitted by plasma-derived medicinal products, the prevalence of parvovirus B19 -specific antibodies is much higher in groups receiving blood products and clotting factors than in other groups^37^. In general, transmission of blood-borne infections via plasma-derived medicinal products occur because of incomplete elimination of virus and in the case of Parvovirus B19 the resistance of the virus to most viral inactivation procedures^38,39^.

NAT(Nucleic Acid Testing) screening for parvovirus B19 is the current universal screening of donated blood^40^. This method cannot detect new or emerging viruses, so it may not be totally effective at preventing the parvovirus B19 transmission. Execution of screening with higher sensitivity would result in a huge waste of blood components^41^. Thus, samples collected for blood transfusion should be screened for parvovirus B19 virus using IgM and IgG ELISA and donors with persistent IgG anti-parvovirus B19 virus could be considered as parvovirus B19 Safe^42^. Technical methods like PCR or the use of small-pore-size nanofiltration can lead to other option for making blood products parvovirus B19 virus safe^43,44^. According to the last WFH annual survey report (2016), only 306 hemophilia patients have been recognized today (although it is expected to be higher)^45^. It should be noted that our finding is subject to some limitations such as small sample size but to the best of our knowledge. In addition, according to patient records, none of them had the history of drug addiction but due to poor information about patients, other sources of transmission for HCV, HBV and HIV (for example sexual transmission) were excluded from this study.

## Conclusion

Our study supports the hypothesis of viral transmission through regular receipt of plasma-derived clotting factors in hemophilia patients. We found a high prevalence of parvovirus B19 and low prevalence of HBV, HCV, HIV, and HTLV-1 among hemophilia patients in Afghanistan. This is the first study investigating five blood-borne viruses in hemophilia patients in Afghanistan. Further studies enrolling a large number of hemophilia patients and collecting detailed diagnostic data would be required to expand on this investigation.

## Material and Methods

### Study population

This cross-sectional descriptive study was conducted between March 2017 and September 2017, 80 hemophilia patients (Type A) in Kabul, Herat, Mazar-i-Sharif and Jalal Abad (Demographic data are summarized in Table 1). Also, this study conducted at Iranian Comprehensive Hemophilia Care Center (ICHCC), Tehran, Iran. The current study also was approved by the Ethical Committee of ICHCC, and the experiment was conducted in compliance with the Declaration of Helsinki. In addition, written informed consent was directly obtained from participants over 18 years old, and for those under the age of 18 years, they were asked to give their verbal assent before the experiment and the informed written consent was obtained from the guardians.

#### Sample collection and serological assay

A 5 ml samples of venous blood was collected from each of the participants into ethylene di-amine tetra-acetic acid (EDTA) sterile tubes. Plasma sample was separated by centrifugation and analyzed for the presence of HIV, anti-HTLV-I, parvovirus B19 (IgG), anti-HCV, and the surface antigen of hepatitis B virus by enzyme-linked immunoassay (ELISA) kits (Dia.Pro, Italy for HCV, HIV, HBV and IBL kit, USA for HTLV-1 and parvovirus B19) according to manufacturer’s instructions using single wells and there was no evidence for cross-reactivity between the tests. All serological tests were performed in Iranian Comprehensive Hemophilia Care Center, Tehran, Iran.

### Molecular detections assayh

The positive samples were further confirmed by polymerase chain reaction (PCR) using specific primers for HCV, HBV & parvovirus B19 (all samples were tested for parvovirus B19). Commercially available extraction kits (NucleoSpin® Dx Virus, Machery-Nagel, Germany) were used for DNA and RNA extraction. Then, DNA samples were kept at −20 °C until further analysis. The HCV RNA was reverse transcribed using a QuantiTect Reverse Transcription Kit (Qiagen, Germany). To amplify a gene fragment of HCV, the HCV cDNA was used as a template in nested PCR method using the following protocol: 25 µL reaction of 10x buffer, MgCl2 (1.5 mM), 0.5-U of Taq DNA polymerase, dNTPs (200 _M), 10 pm of each primer, and 3 µL cDNA. The HBV PCR amplification was carried out in a tube containing 25 µL reaction of 10x buffer, MgCl2 (2 mM), dNTPs (200_M), 10 pm of each primer, 0.5-U of Taq DNA polymerase, and 200 ng DNA.

The PCR amplification was carried out in a tube containing 25 µL reaction of 10x buffer, MgCl2 (1.5 mM), d NTPs (200_M), 10 pm of each primer, 0.5-U of Taq DNA polymerase, and 200 ng DNA in order to confirm the parvovirus b19 infection. Table 3 shows the sequence of primers and PCR conditions for these viruses.

**Table 3:** PCR programs and primers set used for detection of HCV, parvovirus b19 and HBV.

Target	Sequence	PCR Conditions			
Parvo virus B19	F1: GGTTGATTATGTGTGGG R1: ACTGAAGTCATGCTTGG F2: TGTGTGTTGTGTGCAAC R2: CAAACTTCCTTGAAAATG	Step		Temp	Time
		35 cycles	Initial Denaturation	94 °C	5 min
			Denaturation	94 °C	1 min
			Annealing	55 °C	1.5 min
			Extension	72 °C	2 min
			Final Extension	72 °C	10 min
HCV	F1:GAAAGCGTCTAGCCATGGCGTTAGT R1: CTCGCAAGCACCCTATCAGG	Step		Temp	Time
		45 cycles	Initial Denaturation	94 °C	5 min
			Denaturation	94 °C	15 Sec
			Annealing	56 °C	30 Sec
			Extension	72 °C	1 min
			Final Extension	72 °C	10 min
HBV	F1: AGAACATCGCATCAGGACTC R1: CATAGGTATCTTGCGAAAGC F2: AGGACCCCTGCTCGTGTTAC R2: AGATGATGGGATGGGAATAC	Step		Temp	Time
		40 cycles	Initial Denaturation	94 °C	5 min
			Denaturation	94 °C	1 min
			Annealing	55 °C	1 min
			Extension	72 °C	2 min
			Final Extension	72 °C	10 min

### Statistical analysis

Data were collected, entered and analyzed using Fisher’s exact test by SPSS software (version 22, IBM©, USA). Differences in prevalence of HIV, HBV, HCV, HTLV-1 and parvovirus b19 for Socio-demographic characteristics were tested to assess the statistical significance of trends in prevalence of these pathogens over the study period. The 95% confidence interval was calculated based on binomial distribution for HCV, parvovirus B19 and HBV. P value below than 0.05 was regarded as statistically significant.
